# Supplementary material for: Unveiling the Bioactive Potential of the Invasive Jellyfish Phyllorhiza punctata Through Integrative Transcriptomic and Proteomic Analyses
Source: Biomolecules. 2025 Aug 4;15(8):1121. doi: 10.3390/biom15081121 (PMC12383608; doi:10.3390/biom15081121)
Supplement: Supplementary file 1 [file biomolecules-15-01121-s001.zip › Suplementary Material/FileS3/PEPFOLD4/XP_065062068_8896/clusters.html]

 PEP-FOLD clusters


Download archive of all models  here   

|  |  |  |
| --- | --- | --- |
| # | file | sOPEP |
| # cluster 1 - Size: 46 |  |  |
|  | XP\_065062068\_8896-model1 | -16.2365 |
|  | XP\_065062068\_8896-model1.1 | -16.2365 |
|  | XP\_065062068\_8896-model1.2 | -16.1819 |
|  | XP\_065062068\_8896-model1.3 | -16.1819 |
|  | XP\_065062068\_8896-model1.4 | -16.1819 |
|  | XP\_065062068\_8896-model1.5 | -16.1819 |
|  | XP\_065062068\_8896-model1.6 | -16.1819 |
|  | XP\_065062068\_8896-model1.7 | -16.1819 |
|  | XP\_065062068\_8896-model1.8 | -16.1819 |
|  | XP\_065062068\_8896-model1.9 | -16.1819 |
|  | XP\_065062068\_8896-model1.10 | -16.1819 |
|  | XP\_065062068\_8896-model1.11 | -16.1819 |
|  | XP\_065062068\_8896-model1.12 | -16.1819 |
|  | XP\_065062068\_8896-model1.13 | -16.1696 |
|  | XP\_065062068\_8896-model1.14 | -16.1696 |
|  | XP\_065062068\_8896-model1.15 | -16.1696 |
|  | XP\_065062068\_8896-model1.16 | -16.1696 |
|  | XP\_065062068\_8896-model1.17 | -16.1696 |
|  | XP\_065062068\_8896-model1.18 | -16.1696 |
|  | XP\_065062068\_8896-model1.19 | -16.1696 |
|  | XP\_065062068\_8896-model1.20 | -16.1696 |
|  | XP\_065062068\_8896-model1.21 | -16.1696 |
|  | XP\_065062068\_8896-model1.22 | -16.1696 |
|  | XP\_065062068\_8896-model1.23 | -16.1691 |
|  | XP\_065062068\_8896-model1.24 | -16.1691 |
|  | XP\_065062068\_8896-model1.25 | -16.1186 |
|  | XP\_065062068\_8896-model1.26 | -16.1186 |
|  | XP\_065062068\_8896-model1.27 | -16.1186 |
|  | XP\_065062068\_8896-model1.28 | -16.1186 |
|  | XP\_065062068\_8896-model1.29 | -16.1186 |
|  | XP\_065062068\_8896-model1.30 | -16.1186 |
|  | XP\_065062068\_8896-model1.31 | -16.1186 |
|  | XP\_065062068\_8896-model1.32 | -16.1186 |
|  | XP\_065062068\_8896-model1.33 | -16.1186 |
|  | XP\_065062068\_8896-model1.34 | -16.1186 |
|  | XP\_065062068\_8896-model1.35 | -16.1186 |
|  | XP\_065062068\_8896-model1.36 | -16.1186 |
|  | XP\_065062068\_8896-model1.37 | -16.1186 |
|  | XP\_065062068\_8896-model1.38 | -16.1186 |
|  | XP\_065062068\_8896-model1.39 | -16.1186 |
|  | XP\_065062068\_8896-model1.40 | -16.1186 |
|  | XP\_065062068\_8896-model1.41 | -16.1186 |
|  | XP\_065062068\_8896-model1.42 | -16.1186 |
|  | XP\_065062068\_8896-model1.43 | -16.1186 |
|  | XP\_065062068\_8896-model1.44 | -16.1186 |
|  | XP\_065062068\_8896-model1.45 | -16.0972 |
| # cluster 2 - Size: 16 |  |  |
|  | XP\_065062068\_8896-model2 | -16.2256 |
|  | XP\_065062068\_8896-model2.1 | -16.2256 |
|  | XP\_065062068\_8896-model2.2 | -16.2256 |
|  | XP\_065062068\_8896-model2.3 | -16.162 |
|  | XP\_065062068\_8896-model2.4 | -16.162 |
|  | XP\_065062068\_8896-model2.5 | -16.162 |
|  | XP\_065062068\_8896-model2.6 | -16.162 |
|  | XP\_065062068\_8896-model2.7 | -16.162 |
|  | XP\_065062068\_8896-model2.8 | -16.1478 |
|  | XP\_065062068\_8896-model2.9 | -16.1478 |
|  | XP\_065062068\_8896-model2.10 | -16.1478 |
|  | XP\_065062068\_8896-model2.11 | -16.14 |
|  | XP\_065062068\_8896-model2.12 | -16.12 |
|  | XP\_065062068\_8896-model2.13 | -16.12 |
|  | XP\_065062068\_8896-model2.14 | -16.12 |
|  | XP\_065062068\_8896-model2.15 | -16.0849 |
| # cluster 3 - Size: 38 |  |  |
|  | XP\_065062068\_8896-model3 | -16.1582 |
|  | XP\_065062068\_8896-model3.1 | -16.1582 |
|  | XP\_065062068\_8896-model3.2 | -16.1582 |
|  | XP\_065062068\_8896-model3.3 | -16.1402 |
|  | XP\_065062068\_8896-model3.4 | -16.1402 |
|  | XP\_065062068\_8896-model3.5 | -16.1402 |
|  | XP\_065062068\_8896-model3.6 | -16.1402 |
|  | XP\_065062068\_8896-model3.7 | -16.1402 |
|  | XP\_065062068\_8896-model3.8 | -16.1402 |
|  | XP\_065062068\_8896-model3.9 | -16.1402 |
|  | XP\_065062068\_8896-model3.10 | -16.1402 |
|  | XP\_065062068\_8896-model3.11 | -16.1402 |
|  | XP\_065062068\_8896-model3.12 | -16.1402 |
|  | XP\_065062068\_8896-model3.13 | -16.1402 |
|  | XP\_065062068\_8896-model3.14 | -16.1402 |
|  | XP\_065062068\_8896-model3.15 | -16.1402 |
|  | XP\_065062068\_8896-model3.16 | -16.1402 |
|  | XP\_065062068\_8896-model3.17 | -16.1402 |
|  | XP\_065062068\_8896-model3.18 | -16.135 |
|  | XP\_065062068\_8896-model3.19 | -16.135 |
|  | XP\_065062068\_8896-model3.20 | -16.1346 |
|  | XP\_065062068\_8896-model3.21 | -16.1346 |
|  | XP\_065062068\_8896-model3.22 | -16.1346 |
|  | XP\_065062068\_8896-model3.23 | -16.1346 |
|  | XP\_065062068\_8896-model3.24 | -16.1268 |
|  | XP\_065062068\_8896-model3.25 | -16.1268 |
|  | XP\_065062068\_8896-model3.26 | -16.1131 |
|  | XP\_065062068\_8896-model3.27 | -16.1096 |
|  | XP\_065062068\_8896-model3.28 | -16.1096 |
|  | XP\_065062068\_8896-model3.29 | -16.1096 |
|  | XP\_065062068\_8896-model3.30 | -16.1096 |
|  | XP\_065062068\_8896-model3.31 | -16.1096 |
|  | XP\_065062068\_8896-model3.32 | -16.1096 |
|  | XP\_065062068\_8896-model3.33 | -16.1096 |
|  | XP\_065062068\_8896-model3.34 | -16.088 |
|  | XP\_065062068\_8896-model3.35 | -16.088 |
|  | XP\_065062068\_8896-model3.36 | -16.088 |
|  | XP\_065062068\_8896-model3.37 | -16.0768 |
|  |  |  |
